# Revision Distal Bicep Repair in the Setting of Heterotopic Ossification: Case Study

**DOI:** 10.1002/ccr3.71874

**Published:** 2026-01-22

**Authors:** Aidan M. Brikho, Joseph J. Abbo, Matthew J. Yousif

**Affiliations:** ^1^ College of Osteopathic Medicine Touro College of Osteopathic Medicine, Middletown Middletown New York USA; ^2^ Yousif Orthopedics Troy Michigan USA; ^3^ College of Literature, Science, and the Arts University of Michigan Ann Arbor Michigan USA

**Keywords:** Achilles tendon allograft, distal bicep tendon, heterotopic ossification, orthopedic surgery, tendon reconstruction

## Abstract

Heterotopic ossification (HO) following distal biceps tendon repair is an uncommon but potential complication. This may present pain, limited range of motion, or sensory changes due to nerve compression. This case study reports the case of a patient who developed significant functional limitation after primary distal biceps tendon repair complicated by heterotopic ossification. Radiographs confirmed extensive ossification surrounding the radial tuberosity. Plain radiographs were obtained to confirm the presence and maturity of the HO; a CT scan was performed to delineate the extent and proximity to the neurovascular bundle for operative planning. Surgical revision was performed using an Achillies tendon allograft to reconstruct the distal biceps tendon while excising the ossified tissue. Postoperatively, the patient experienced marked improvement in motion and resolution of pain. No recurrence was observed at follow‐up. This case emphasizes the importance of early recognition of heterotopic ossification after tendon repair and demonstrates that timely revision with an Achillies allograft can effectively restore elbow function and minimize recurrence.

## Key Clinical Message

Heterotopic ossification (HO) is a rare but significant complication following a distal biceps tendon repair. It can lead to functional impairment and soft tissue pain. Early recognition, prevention, and reconstruction with an Achilles tendon allograft can restore function in cases of failed primary repair.

## Graphical abstract

Radiographic and intraoperative findings demonstrating heterotopic ossification following distal biceps tendon repair.

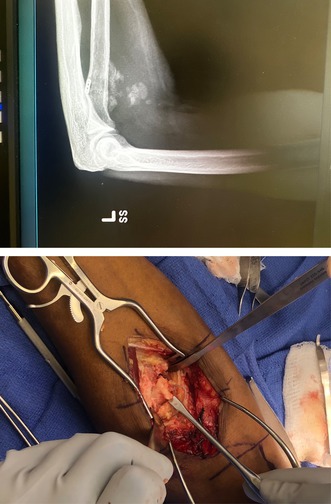

## 1 Introduction

Ruptures of the distal biceps brachii tendon at its radial tuberosity attachment are relatively uncommon injuries, predominantly occurring in men during their third or fourth decades of life and commonly involving their dominant arm subjected to an acute eccentric loading force. In the United States, distal biceps tendon injuries are reported at a rate of 2.55 cases per 100,000 patient‐years [1]. Surgical repair remains the standard treatment for active individuals, as it restores both strength and function [2]. However, surgical repair poses its own risk, with potential complications such as neurovascular injury, heterotopic ossification (HO), surgical site infections, elbow stiffness, and re‐rupture. HO is a rare but severe complication that can present as erythema, swelling, pain, restricted joint mobility, or neurovascular compression, though it may also be asymptomatic [3, 4].

In the case presented, HO following index distal biceps tendon repair resulted in lateral antebrachial dysesthesia with potential AIN and PIN neuropathy, significantly impacting the patient's functional recovery, evidently due to the severity of this patient's HO. Early detection and intervention are critical for mitigating this surgical complication. HO occurs infrequently, with symptomatic postoperative cases observed in only 1.3% of patients in a study of 970 individuals undergoing double‐incision biceps repair [5]. HO involves abnormal bone formation within soft tissues, which can lead to pain, swelling, restricted movement of the affected joint and dysesthesia from nerve compression from attritional wear of local soft tissue and tendons [6]. While asymptomatic cases are more common, those presenting with nerve compression or joint dysfunction can severely hinder recovery [1]. These functional, symptomatic complications necessitated reconstruction using an Achilles tendon allograft. By analyzing this patient's unique clinical presentation and reviewing existing literature, this paper aims to enhance understanding of the risk factors, presentations, and management strategies for heterotopic ossification following distal biceps tendon repairs. The following report describes a patient who developed severe HO after distal biceps tendon repair which ultimately led to revision surgery using an Achilles tendon allograft.

## 2 Case History and Clinical Examination

A 47‐year‐old right handed dominant male with no significant comorbidities, initially presented for evaluation following primary distal biceps tendon repair performed for an acute eccentric loading injury of the dominant arm. The post operative course was remarkable at first, 6–8 weeks after surgery the patient noticed gradual pain, decreased range of motion of active flexion/supination at the elbow and swelling described specifically as firm, non‐fluctuant fulness located at the center of the antecubital fossa, no gross findings noted on the contralateral limb. The patient reported numbness and tingling along the lateral forearm, which is consistent with involvement of the lateral antebrachial cutaneous nerve (LABCN) and described weakness when performing elbow flexion and forearm supination.

Upon presentation, the patient had visible swelling over the antecubital fossa as well as palpable firmness in the region over the repair. The patient was restricted with active 30°–90° and passive 20°–100° end range of motion. A Disabilities of the Arm, Shoulder, and Hand score (DASH) was obtained and valued at 45. Upon sensory testing, dysesthesia was demonstrated along the LABCN distribution. The patient also showed weakness in pinch and finger extension, which raised the concern of possible anterior interosseous nerve (AIN) and posterior interosseous nerve (PIN) neuropathy.

**FIGURE 1:**
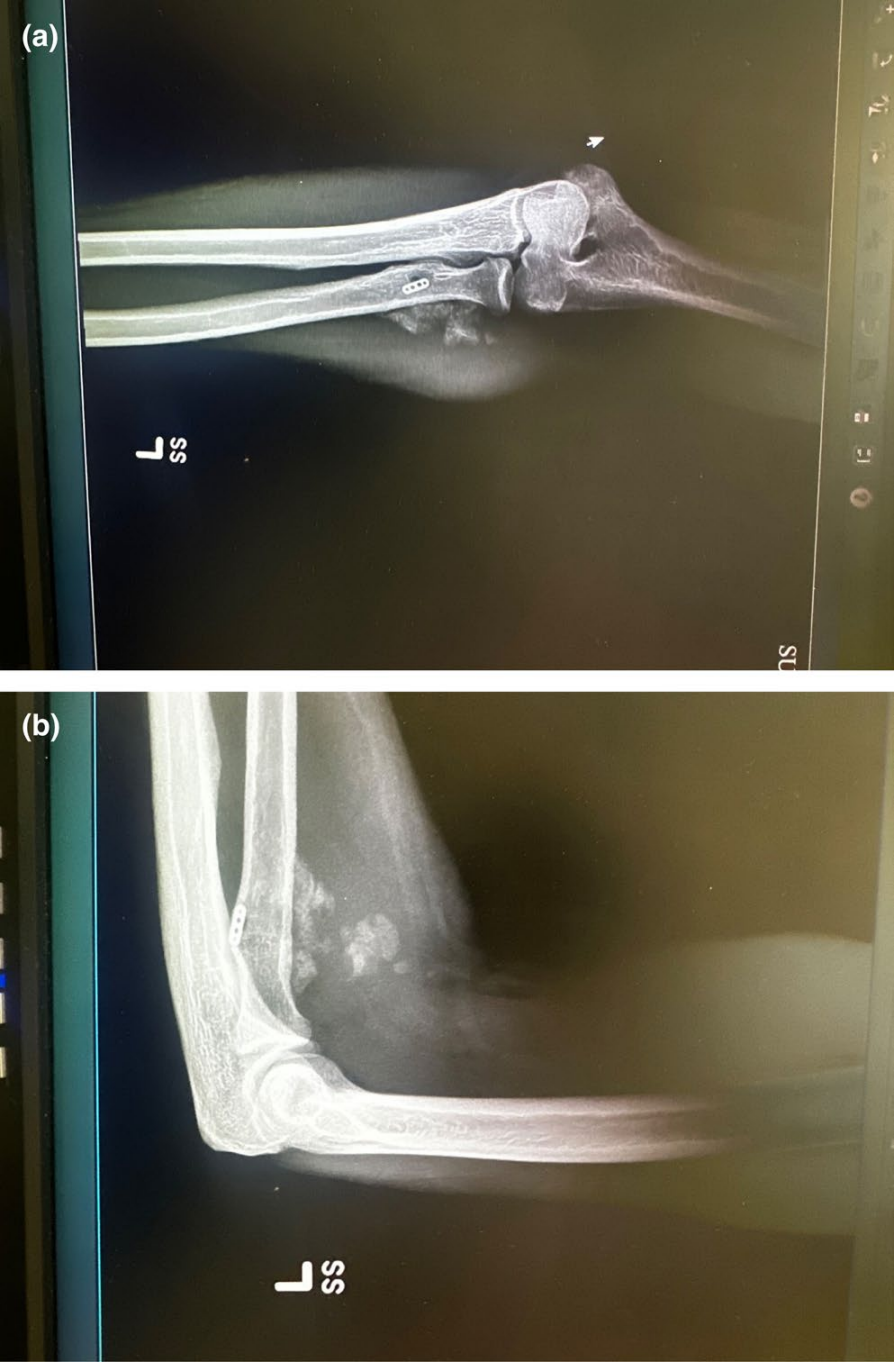
(a) Preoperative anterior to posterior radiograph of left elbow, demonstrating heterotopic ossification after primary repair of the distal biceps tendon. EndoButton is in an appropriate position sitting 3 × 2 × 3 cm within the radial tuberosity. (b) Preoperative lateral radiograph image of left distal bicep showing hetero ossification superior to the radial tuberosity. EndoButton is well aligned.

Plain radiographs of the left elbow (Figure 1a) revealed heterotopic ossification at the site of primary repair and included bony bridging across soft tissue planes. Subsequent Computed Axial Tomography (CT scan) to the affected area without contrast was performed, demonstrating heterotopic ossification around the radial head and volar soft tissue, further highlighting the extent of the HO surrounding the previous repair, confirming the cause of the patients' pain and restricted mobility. The HO mass appeared to impinge on nearby neurovascular structures. These findings are correlated to the symptoms described by the patient. The diagnosis of heterotopic ossification at the insertion of the distal biceps was made. This was significant enough to warrant surgical revision.

## 3 Differential Diagnosis, Investigations, and Treatment

Heterotopic ossification (HO) around the elbow is classified into three categories by Hastings and Graham, with class III being the most severe, characterized by bony ankylosis and complete loss of motion [7]. the development of HO involves a series of complex cellular and molecular events. Initially, localized tissue damage triggers inflammation, resulting in the release of pro‐inflammatory cytokines such as interleukin‐1 (IL‐1) and tumor necrosis factor‐alpha (TNF‐α). These cytokines recruit mesenchymal stem cells to the injury site [8]. Upon recruitment, these stem cells differentiate into osteoblasts, a process driven by the activation of key signaling pathways, including bone morphogenetic protein (BMP) and transforming growth factor‐beta (TGF‐β), ultimately leading to ectopic bone formation [9]. In distal biceps tendon repair, surgical disruption of soft tissues, coupled with the patient's postoperative inflammatory response, establishes conditions conducive to HO formation. Over time, ectopic bone growth may compress nearby structures, such as the lateral antebrachial nerve, resulting in dysesthesia, pain, and restricted mobility, as demonstrated in this case. Prompt identification and management of HO are essential to minimizing complications and ensuring optimal functional recovery [1, 2].

The clinical presentation of the patient matched the pathophysiology that is explained above. It was decided to pursue surgical management, which went as follows.

### 3.1 Surgical Intervention

**FIGURE 2:**
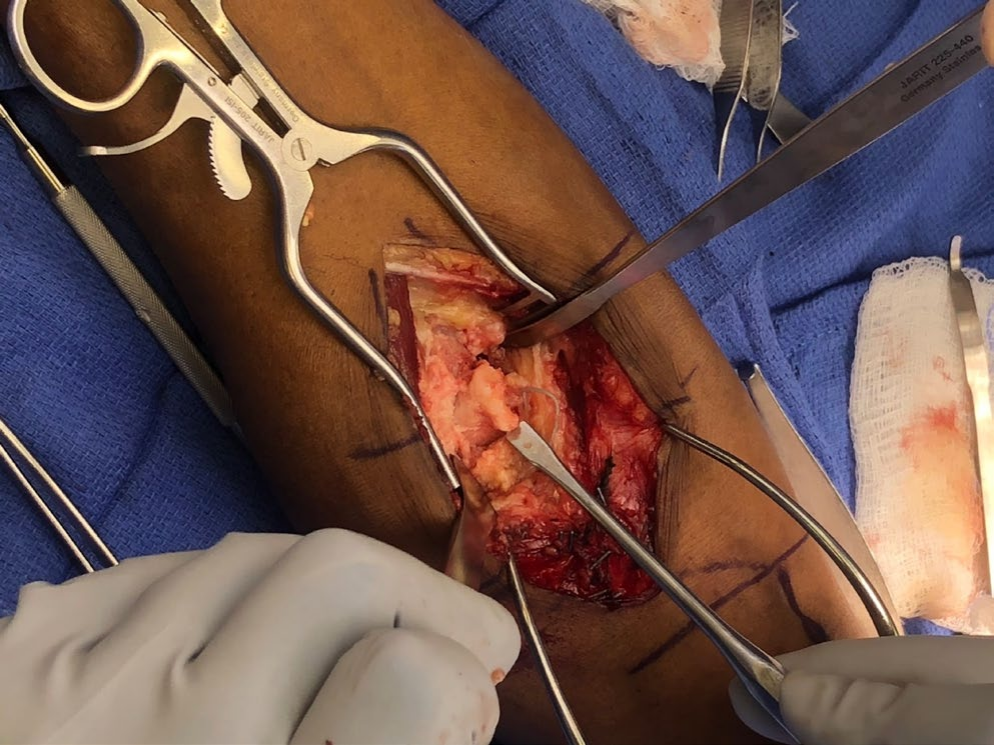
Intraoperative images of left distal bicep tendon showing significant heterotopic ossification prior to debridement.

**FIGURE 3:**
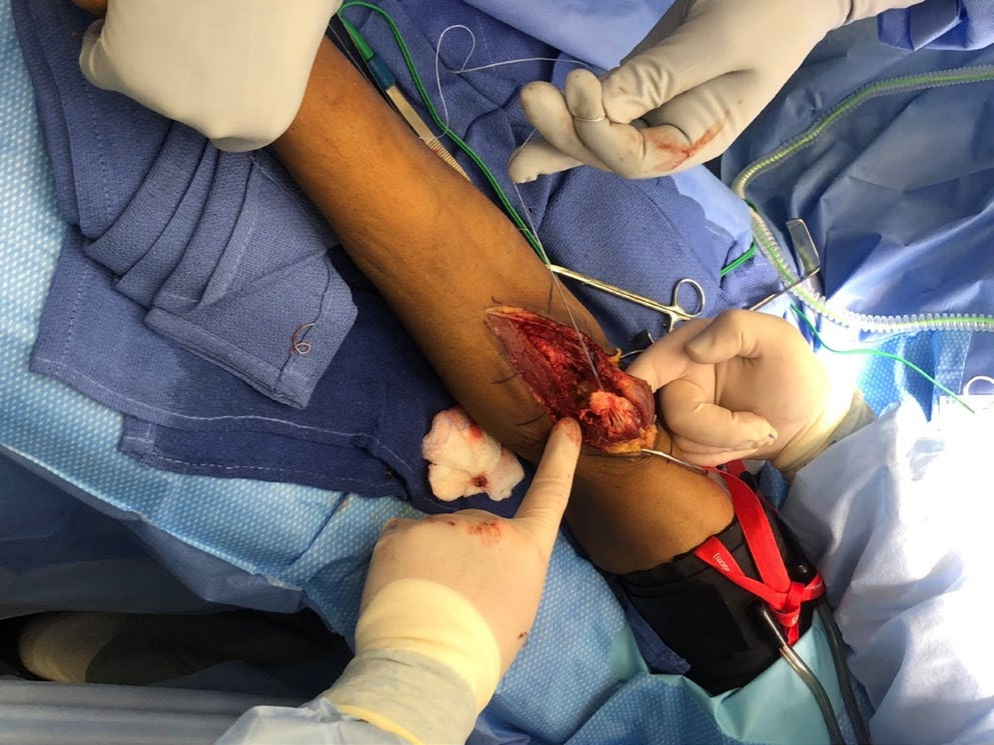
Intraoperative image showing 5 mm of the distal biceps stump prior to fixation.

**FIGURE 4:**
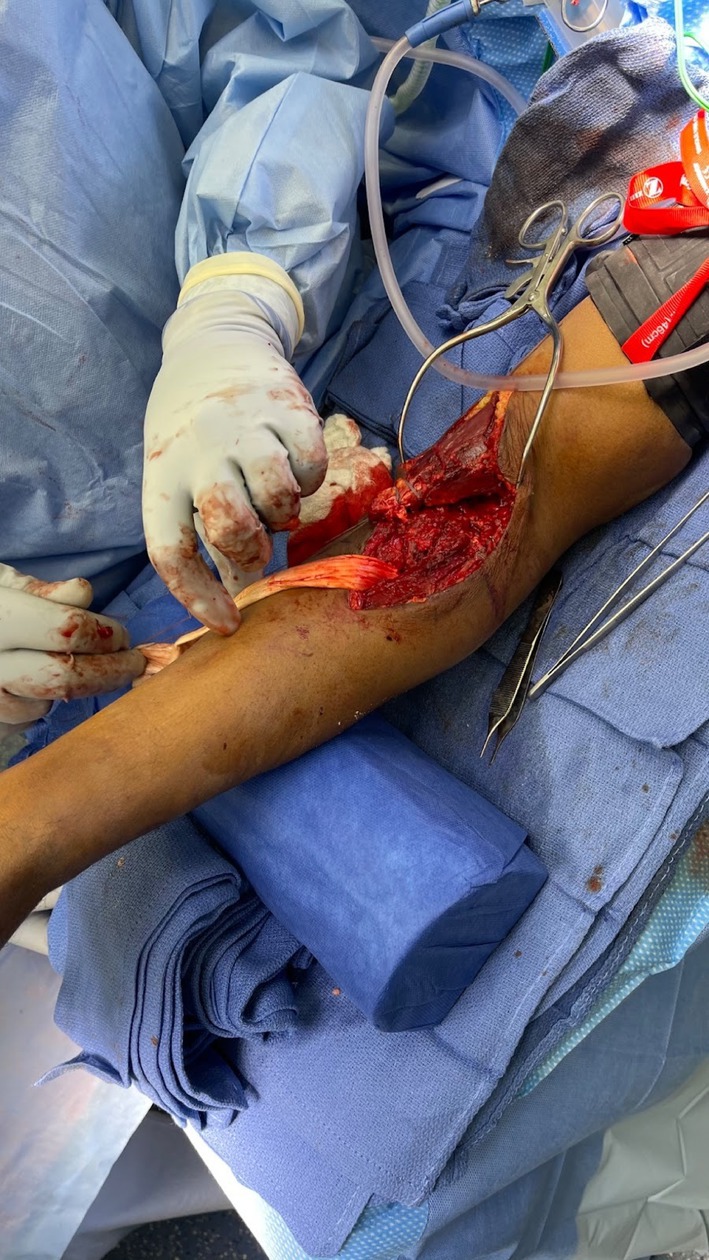
Implementation of Achilles tendon allograft into radial tuberosity in preparation for fixation between distal biceps stump (located proximal).

**FIGURE 5:**
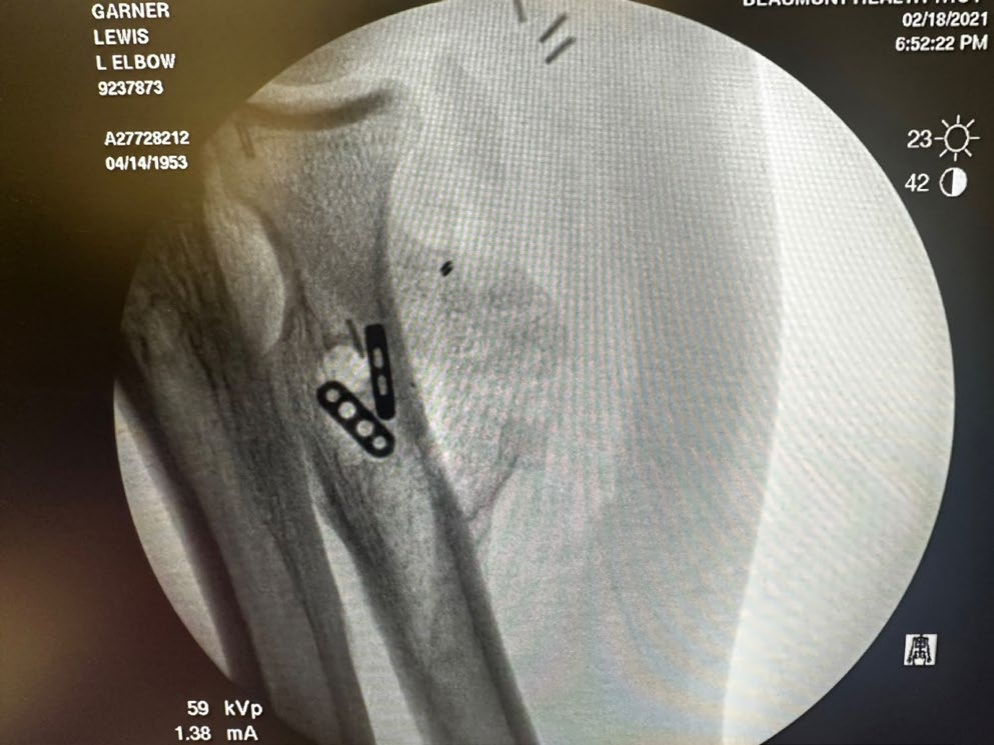
Intraoperative radiograph showing perfect alignment of secondary and primary fibertak stitch with significant decrease of heterotopic ossification.

The patient had previously undergone primary distal biceps tendon repair using a single incision technique, which subsequently developed HO at the radial tuberosity insertion site, resulting in his pain and restrictive motion. The previous button was in good alignment (Figure 1a,b). The Patient was placed supine on a standard operating table. With a vascular hand attachment added to the table. The patient was prepped appropriately. Prophylactic intravenous antibiotics were administered prior to incision. A single sterile tourniquet was applied and utilized intermittently for visualization during the procedure. A single volar approach was used. One longitudinal incision was made along the volar radial third of the forearm, crossing the antecubital crease and extending about 10 cm proximally. Sharp dissection was carried out through skin and subcutaneous tissue. Medial and lateral skin flaps were made and sutured down as retracting tag stitches to the skin. Additionally, the skin was irrigated to avoid any bacterial infection and resolve bleeding control. The lateral antebrachial cutaneous nerve (LABCN) was encountered with significant dense heterotopic ossification to this area. A careful neurolysis was performed of the LABCN, approximately 3 × 3 × 3 cm of heterotopic ossification was excised using an osteotome and rongeur while protecting the nerve (Figure 2). At this point, the biceps tendon stump was identified and measured at 0.5 cm of stump length present (Figure 3). Previous suture from the primary repair was removed meticulously. A fiber link suture was circumferentially placed around the stump in a whip stich fashion using a FiberLoop suture and the free needle at the end of the FiberLink (Arthrex, Naples, FL) suture to the end of the tendon, this served as a traction stitch to mobilize the anterior and posterior surface. Blunt dissection was then performed anterior to posterior to release all adhesions. The biceps was mobilized proximally while protecting the neurovascular bundle which was isolated using a Penrose drain for gentle traction. Attention was then turned to the radial tuberosity; the prior cortical fixation site was exposed, debrided, and irrigated. Cancellous Bone graft from cadaveric tissue was placed to restore native contour. An Achilles tendon allograft was prepared and tubularized to approximately 7 mm in diameter. A FiberLoop (Arthrex, Naples, FL) suture was placed around the distal 4 cm of the Achilles tendon allograft in proportion to fixation of the bone (Figure 4). The previous cortical button site was utilized; an Arthrex Beath Pin (Arthrex, Naples, FL) and cannulated drill bit were used to re‐establish the tunnel. The Achilles tendon allograft was secured using a cortical button, which was deployed and confirmed in proper position using intraoperative fluoroscopy (Figure 5). The graft was tensioned proximally, and the stump was passed longitudinally through the graft and oversewn using 4 simple 0 Vicryl sutures to achieve stable layered tendon to the graft interface, approximated to be 5 cm of tendon to graft overlap. After hemostasis was maintained, the wound was closed in a primary fashion from deep to superficial. The elbow was immobilized at 90° of flexion in a posterior splint for 2 weeks. He was then transferred to the post anesthesia care unit.

### 3.2 Rehabilitation and Plan

A single dose of localized radiation therapy (7 Gy) was administered 24 h post operatively as a prophylactic against reoccurring heterotopic ossification. Interval Naproxen 50 mg was administered and taken twice daily for 6 weeks as HO prophylaxis. Following surgical intervention, the patient was placed in a Breg T‐Scope Elbow Brace position at 90° of flexion with the forearm at neutral rotation. For the first 2 weeks post operatively, the brace is to remain locked at 90°. While in physical therapy, it is recommended for shoulder pendulum exercises as well as gentle hand, rest, and finger movements to maintain cardiovascular circulation [10]. Between the second and fourth week, the brace was gradually adjusted to allow 45°–100° of flexion. While in physical therapy it is advised for passive elbow motion while avoiding any biceps contraction [11]. Between weeks four and six, the brace was unlocked, permitting 30°–120° of mobility. Under therapist supervision, the patient is allowed to begin active, assisted exercises of the affected arm. Soft‐tissue massage and scar mobilization were incorporated to improve flexibility and minimize adhesions [11]. At 6 weeks postoperative, the brace was unlocked to full mobility and worn only during high‐risk activities. While in therapy, the patient began active exercises under therapist supervision. During the 12th week, strengthening began with light, isotonic release, emphasizing endurance and proper perceptive control [10]. At 3 months, the brace was discontinued, and the patient progressed as tolerated. At the patient 6‐month postoperative visit, full mobility from 0° to 140° of active range of motion, 5/5 strength upon supination and flexion in comparison to the contralateral unaffected side, complete resolution of previous dysesthesia and a DASH score of 7. A final 1 year post operative visit was documented; the patient has resumed normal activity with no complaints of pain.

## 4 Conclusion and Results

Heterotopic ossification (HO) continues to pose a challenge for postoperative complications, particularly in cases of two‐incision distal biceps tendon repair. This case study highlights the severity of an HO causing dysesthesia and soft tissue pain, eventually leading to a secondary procedure of biceps reconstruction using an Achilles tendon allograft, after failure of the primary repair. Using a literature review and surgical intervention, this report emphasizes the importance of early detection and effective surgical techniques. Also included in this study are many proactive preventive measures such as nonsteroidal anti‐inflammatory medications and radiation therapy to reduce HO recurrence. Both offer promising approaches for mitigating HO formation in the future [12]. Further research is still needed to establish protocols for high‐risk patients which emphasize the need for progressive treatment and prevention strategies. By enhancing surgical techniques and using evidence‐based measures to prevent heterotopic ossification, clinicians can have improved patient outcomes.

Postoperatively, the patient was able to demonstrate restoration of their elbow flexion strength and had a resolution of dysesthesia in the lateral antebrachial cutaneous nerve distribution. Rehabilitation after surgery was initiated and at the short‐term follow‐up it was shown that the range of motion and functional use of the arm had significantly improved. Continued monitoring was deemed the appropriate course of action to assess for the recurrence of HO.

## 5 Discussion of Literature Review

Heterotopic ossification represents a significant postoperative complication; as seen in our current case, extensive ossification was present and led to significant nerve and soft tissue symptoms of the distal biceps tendon with retraction. The literature stresses that HO is driven by inflammatory processes and trauma to the soft tissue, which can trigger ectopic bone formation through mesenchymal stem cell differentiation [9]. The risk of HO is heightened in situations involving prolonged immobilization, excessive soft tissue trauma, or genetic predispositions. One such genetic factor is mutations in the ACVR1 gene, which encodes a BMP receptor that binds serine/threonine to transform the growth factor. These mutations are strongly associated with fibrodysplasia ossificans progressiva (FOP), a rare disorder marked by widespread and progressive HO [13]. ACVR1 mutations cause the BMP receptor to become constitutively active, even without BMP ligand binding. This hyperactivity disrupts the normal regulation of BMP signaling, creating a microenvironment favorable to uncontrolled bone formation in soft tissues. While FOP is the most well‐documented manifestation of ACVR1 mutations, similar genetic mechanisms are thought to contribute to localized HO following trauma or surgical interventions [13]. HO most commonly occurs due to several factors such as; the use of a two‐ incision technique during initial repair, inadequate irrigation during intraoperative reaming of the bone and pre‐disposed patient factors. The two‐incision approach has been associated with increased soft tissue disruption, which can promote ectopic bone formation in the surrounding tissues. Inadequate irrigation during the reaming process may leave residual bone debris in the soft tissue, further contributing to the inflammatory cascade that triggers HO Additionally, certain patient factors, such as a heightened inflammatory response and genetic predisposition can increase the risk of heterotopic bone formation. These combined surgical and biological factors necessitate a need for this paper to highlight the importance of meticulous surgical technique and perioperative management to minimize the incidence of this complication [14]. Our case highlights the severity of HO, evidenced through the pre operative CT and magnetic resonance imaging (MRI), both of which confirm significant ossification and structural tearing of the tendon. Reconstruction required an Achilles tendon allograft. This allograft was chosen and supported by literature emphasizing its structural integrity and compatibility for tendon repair, especially in cases where the live tissue is insufficient. Achilles tendon allografts are well known for their excellent mechanical properties and compatibility; the Achilles tendon offers an alternative to autografts and avoids donor site morbidity. Studies have demonstrated that Achilles tendon allografts provide robust mechanical strength, biological compatibility, and ease of surgical manipulation, making them an optimal graft choice in revision of the distal bicep where tissue quality is compromised by HO [15]. The grafts' large structure can withstand mechanical demands effectively, while minimizing surgical trauma. In our case, the use of the Achilles tendon allograft allowed for surgical reconstruction of the distal biceps while decreasing patient morbidity. This case review aims to highlight the severity of HO and provide insights into decreasing its recurrence. Although conservative measures are an option in HO treatment in the setting of a distal bicep tendon repair, optimizing surgical techniques and using pharmacological interventions concomitantly such as Nonsteroidal anti‐inflammatory drugs (NSAIDs) or localized radiation, both crucial in reducing ectopic bone formation [5, 12]. The findings highlight the importance of early diagnosis, effective surgical strategies, and targeted prevention to improve patient outcomes. Showcasing this unique case and its management contributes to the literature addressing HO. related complications in reconstructive surgeries.

NSAIDs, specifically naproxen and indomethacin, are well‐established in preventing HO. They both can inhibit prostaglandin synthesis, which promotes ectopic bone formation from the inflammatory cascade. Naproxen has gained popularity because it effectively reduces HO formation after surgical intervention. While NSAIDs stay as the best choice of prophylaxis, exploring additional strategies is important to reducing HO risk, especially in complex cases or when NSAIDs are contraindicated. Radiation therapy is an emerging treatment for HO prevention. Studies have shown that a single postoperative dose of 7 Gy, in combination with a short course of NSAIDs, significantly decreases HO formation. While this protocol has been extensively studied in total hip arthroplasty, its application to other joints, including the elbow, is promising [12]. Radiation therapy offers an effective preventative measure by targeting it at a cellular level. Bisphosphonates are another potential treatment option, primarily known for their role in managing metabolic bone diseases. These agents work by inhibiting osteoclast bone resorption, which can disrupt the processes that contribute to ectopic bone growth [16]. Bisphosphonates are less commonly used for HO. Bisphosphonates are less commonly used for HO but provide an alternative for patients unable to tolerate NSAIDs; it can also be used as a supplementary measure. Future research can be conducted following this to optimize dosing protocols and popularize their role in practice. These approaches aim to reduce recurrence rates and improve outcomes for patients undergoing surgical repair in high‐risk joints, such as the elbow.

## Author Contributions

**Aidan M. Brikho:** writing – original draft, writing – review and editing. **Joseph J. Abbo:** writing – original draft, writing – review and editing. **Matthew J. Yousif:** methodology, supervision, writing – original draft, writing – review and editing.

## Funding

The authors have nothing to report.

## Consent

Written informed consent was obtained from the patient for the publication of this case report and accompanying images.

## Conflicts of Interest

The authors declare no conflicts of interest.

## Data Availability

The data that support the findings of this study are openly available in *American Journal of Sports Medicine* at https://doi.org/10.1177/0363546519890933, reference number [https://doi.org/10.1177/0363546519899933].

## References

[1] J. G. Hart and B. J. MacKay , “Complications in Distal Biceps Tendon Repair and Reconstruction: Incidence and Management Strategies,” Journal of Orthopaedic Research 40, no. 8 (2022): 1345–1353, 10.1016/j.jor.2022.03.003.

[2] NYU Langone Health , “Repair Surgery for Distal Biceps Tendon Rupture,” accessed January 26, 2025, https://nyulangone.org/conditions/distal‐biceps‐tendon‐rupture/treatments/repair‐surgery‐for‐distal‐biceps‐tendon‐rupture.

[3] Cleveland Clinic , “Heterotopic Ossification: Symptoms, Causes, Treatment, and Prevention,” accessed January 26, 2025, https://my.clevelandclinic.org/health/diseases/22596‐heterotopic‐ossification.

[4] EBSCO Research , “Complications in Distal Biceps Tendon Repair,” accessed January 26, 2025, https://research.ebsco.com/c/3nr5oa/viewer/html/k4kj4l6ihr.

[5] H. De Boeck and K. De Smet , “Surgical Complications and Prevention Strategies in Orthopedic Reconstructions,” Acta Orthopaedica Belgica 89, no. 5 (2023): 695–702.38205763

[6] N. Wu , Pinched Nerve in Elbow: Symptoms, Causes, and Treatments (WebMD), accessed January 26, 2025, https://www.webmd.com/pain‐management/pinched‐nerve‐in‐elbow.

[7] H. Hastings, 2nd and T. J. Graham , “The Classification and Treatment of Heterotopic Ossification About the Elbow and Forearm,” Hand Clinics 10, no. 3 (1994): 417–437. 7962148

[8] M. Subramaniam , T. Koike , and R. S. Morrison , “Tumor Necrosis Factor‐Alpha and Interleukin‐1 Beta Mediate Pro‐Inflammatory Cytokine‐Induced Apoptosis in Neurons,” ACS Chemical Biology 2, no. 2 (2007): 91–100, 10.1021/pr060665l.

[9] C. R. Schlieve , S. Soma , T. Iwanaga , R. Aoki , R. Nishinakamura , and A. Kurisaki , “The Role of Immune Regulation in Heterotopic Ossification: Mesenchymal Stem Cell Differentiation and Inflammatory Pathways,” Immunity 50, no. 5 (2019): 1019–1032, 10.1016/j.immuni.2019.03.019.

[10] Massachusetts General Hospital , Rehabilitation Protocol for Distal Biceps Tendon Repair (Mass General Brigham Sports Medicine, 2021), https://www.massgeneral.org/assets/mgh/pdf/orthopaedics/sports‐medicine/physical‐therapy/rehabilitation‐protocol‐for‐distal‐biceps‐repair.pdf.

[11] C. A. Logan , A. Shahien , D. Haber , Z. Foster , A. Farrington , and M. T. Provencher , “Rehabilitation Following Distal Biceps Repair,” International Journal of Sports Physical Therapy 14, no. 2 (2019): 308–317, 10.26603/ijspt20190308. 30997282 PMC6449020

[12] E. E. Pakos , E. J. Pitouli , P. G. Tsekeris , A. Papathanasopoulos , K. S. Stafilas , and T. A. Xenakis , “Prevention of Heterotopic Ossification in High‐Risk Patients With Total Hip Arthroplasty: The Experience of a Combined Therapeutic Protocol,” International Orthopaedics 30, no. 2 (2006): 79–83, 10.1007/s00264-005-0054-y. 16482442 PMC2532069

[13] F. S. Kaplan , M. Xu , P. Seemann , et al., “Classic and Atypical Fibrodysplasia Ossificans Progressiva (FOP): Mutations in the BMP Type I Receptor ACVR1,” Journal of Bone and Mineral Research 24, no. 3 (2009): 475–487, 10.1359/jbmr.090205. 19085907 PMC2921861

[14] R. Patel , “Complications of Distal Biceps Tendon Repair,” accessed January 26, 2025, https://www.drronakpatel.com/wp‐content/uploads/2023/01/11‐Complications‐of‐Distal‐Biceps‐Tendon‐Repair.pdf.

[15] M. Amarasooriya , G. I. Bain , T. Roper , K. Bryant , K. Iqbal , and J. Phadnis , “Complications After Distal Biceps Tendon Repair: A Systematic Review,” American Journal of Sports Medicine 48, no. 12 (2020): 3103–3111, 10.1177/0363546519899933. 32091914

[16] P. Khoueir , R. Blackwell , and J. Rasouli , “Bisphosphonates as a Therapeutic Option for Heterotopic Ossification: A Review of Current Evidence,” Quill Scope 14 (2021): 35–42.

